# Exploring the Views of Barbers and Stylists on the Acceptability of Delivering Community-Based Interventions to Promote COVID-19 Testing and Vaccination in South Carolina

**DOI:** 10.3390/vaccines12091011

**Published:** 2024-09-03

**Authors:** Paddington T. Mundagowa, Sachi Vora, Fatima Seck, Neal Dhankhode, Kwame S. Sakyi, Mufaro Kanyangarara

**Affiliations:** 1 Department of Epidemiology and Biostatistics, Arnold School of Public Health, University of South Carolina, Columbia, SC 29201, USA; 2 Public and Environmental Wellness, School of Health Sciences, Oakland University, Rochester, MI 48309, USA

**Keywords:** COVID-19 testing and vaccination, barbers and stylists, community health, intervention acceptability, South Carolina

## Abstract

Background: The COVID-19 pandemic has underscored the need for effective community-based interventions to promote disease prevention and reach high-risk, underserved communities. Trusted community leaders like barbers and stylists may serve as effective conduits for intervention implementation. This study aimed to explore the perceived acceptability of an intervention to promote COVID-19 testing and vaccination delivered by barbers in South Carolina. Methods: We conducted exploratory in-depth interviews to ascertain barbers’ and stylists’ perceptions and identify potential barriers and facilitators. Data analysis used a deductive coding approach to identify themes and was guided by the Theoretical Framework of Acceptability. Results: Sixteen participants were interviewed. Participants expressed positive reactions towards the interventions. Acceptability was influenced by strong trust relationships with clients, perceived community influence, self-efficacy in providing the intervention, and a shared sense of responsibility for community health. However, potential barriers included declining public concern about COVID-19, vaccine hesitancy, and limited COVID-19 knowledge among barbers and stylists. Participants emphasized the need for training and incentives for effective and sustained intervention delivery. Conclusions: Barbers and stylists are well-positioned to promote COVID-19 testing and vaccination due to their trusted roles and community influence. Given the complacency from the waning perceived COVID-19 threat and the historical mistrust in health interventions, vaccine hesitancy must be addressed through supportive communication strategies.

## 1. Introduction

The coronavirus disease of 2019 (COVID-19) pandemic has had a significant public health impact, resulting in over 776 million cases and 7 million related deaths globally [1]. The pandemic has highlighted long-standing health disparities among the racial and ethnic minority groups in the United States [2]. African Americans and other minority groups have been disproportionately affected and have experienced significantly higher rates of infection, hospitalizations, and deaths [3]. COVID-19 testing and vaccination are critical for controlling the spread of the virus and preventing further morbidity and mortality; however, uptake among racial and ethnic minority groups has been limited [4]. COVID-19 health disparities are influenced by a complex interplay of social, environmental, economic, and cultural factors, including mistrust in medical institutions, socioeconomic challenges, concerns about vaccine safety and efficacy, and limited access to healthcare [5,6]. Institutional mistrust, rooted in a history of medical exploitation and mistreatment, has fostered vaccine hesitancy and a reluctance to engage with healthcare services [7,8]. Furthermore, misinformation about COVID-19 vaccines has proliferated, further hindering vaccination efforts in these communities [9]. Culturally tailored strategies are needed to address these challenges, improve COVID-19 testing and vaccination, and address COVID-19 health disparities.

Community-based interventions have emerged as an effective solution to improve COVID-19 testing and vaccination uptake among high-risk, underserved populations [10,11]. These interventions leverage the trust and influence of local community figures to disseminate information about COVID-19 and promote COVID-19 prevention, testing, and vaccination [12]. Their effectiveness relies heavily on the degree of trust that community members have in the figures delivering the message [12,13]. Trust, in this context, is defined as “the willingness of a party to be vulnerable to the actions of another party based on the expectation that the other will perform a particular action important to the trustor, irrespective of the ability to monitor or control the other party” [14]. Partnering with trusted community figures can bridge the gap between public health officials and communities by increasing COVID-19 health literacy, addressing COVID-19 vaccine hesitancy, influencing the uptake of preventive measures, and reaching community members who may not have access to or receive healthcare services.

Barbers and stylists, as trusted community figures, are uniquely positioned to serve as conduits for public health messaging [15,16]. Their frequent and personal interactions with clients provide an excellent opportunity to disseminate accurate health information, address vaccine hesitancy, and promote COVID-19 testing and vaccination. Moreover, barbershops and salons are deeply embedded in the social fabric of African American communities, not only providing hair and beauty services but also serving as community pillars for social interaction and information exchange. These establishments provide a culturally relevant and trusted environment to effectively reach underserved populations with health promotion interventions [15,16,17]. Previous public health initiatives have leveraged partnerships with barbershops and beauty salons to train providers, improve health literacy, initiate client referrals to healthcare providers, promote positive attitudes, and provide informal mental health support [16,18,19,20,21,22,23,24]. These initiatives have focused on conditions supporting the management of chronic conditions such as hypertension, diabetes, cancer, mental health conditions, and HIV/AIDS. In the context of COVID-19, several public health initiatives have partnered with barbers and stylists to address COVID-19 vaccine hesitancy and misinformation and to improve COVID-19 testing and vaccination in marginalized communities [8,25]. The success of these interventions has been attributed to the cultural relevance and trust established in barbershops and salons, which are seen as safe and familiar environments [16,25]. Their established relationships with clients, community influence, and trust can enhance the credibility and acceptance of health interventions. Despite the growing interest in leveraging the trusted roles of barbers and stylists to increase the reach and effectiveness of COVID-19 prevention efforts, studies exploring the acceptability of barbershop- and salon-based interventions from the perspective of barbers and stylists are lacking.

Intervention acceptability is “a multifaceted construct reflecting the extent to which people delivering or receiving an intervention consider it appropriate, based on anticipated or experienced emotional and cognitive responses to the intervention” [26]. By examining the attitudes, perceptions, and responses of both the implementers and recipients to the intervention and its delivery, interventions can be tailored to be culturally appropriate and aligned with community needs and values, enhancing the likelihood of effectiveness. While acceptability alone does not guarantee intervention effectiveness, acceptable interventions are more likely to be implemented, scaled up, and sustained over time [27]. This study aimed to determine the prospective acceptability of barbers and stylists delivering interventions to promote COVID-19 testing and vaccination within community settings in South Carolina. We used qualitative methods to understand the factors influencing acceptability and explore the anticipated barriers and facilitators to intervention implementation. Our findings provide actionable recommendations to inform the development, implementation, and evaluation of an acceptable, culturally tailored COVID-19 prevention intervention delivered in barbershops and salons in South Carolina.

## 2. Materials and Methods

### 2.1. Conceptual Framework

Several theoretical frameworks serve as valuable tools in understanding the factors shaping the acceptability of health interventions. Studies on the acceptance of COVID-19 interventions have extensively used models such as the Health Belief Model [28], the Cognitive–Affective–Normative Model [29], the Theoretical Domains Framework, and the Theoretical Framework of Acceptability [26,30]. To conceptualize the acceptability of barbers and stylists promoting COVID-19 testing and vaccination, we selected the Theoretical Framework of Acceptability (TFA) [26]. The TFA model was chosen because it is more geared toward acceptability than the others because it allows for exploring before, during, and after an intervention, whilst the others are broad health behavior frameworks. In addition, TFA enables a more comprehensive intervention acceptability assessment and provides opportunities for iterations to intervention guided by specific domains [31]. The TFA has been widely used to understand intervention acceptability in the context of HIV prevention and treatment [32], diabetes prevention and management [33], kidney disease management [34], mental health promotion [35,36], and more recently, COVID-19 prevention measures [37,38]. By delineating constructs such as affective attitude, burden, and self-efficacy, the TFA allows for the exploration of the emotional and cognitive responses underlying intervention acceptability throughout the implementation process (before, during, and after intervention). 

### 2.2. Study Design

We employed an exploratory qualitative study design to capture the breadth and depth of barbers’ and stylists’ perspectives. This study is part of a project to partner with barbershops and salons to reduce COVID-19 health disparities among high-risk, underserved populations in South Carolina by improving their access to and uptake of COVID-19 testing and vaccination.

### 2.3. Study Participants and Setting

From September to November 2023, in-depth interviews were conducted in four counties in SC—Lexington, Orangeburg, Richland, and Sumter. These counties were selected because they had a higher demographic of underserved minority populations [39] and lower testing and vaccination rates [40]. Barbershops and salons serving communities with higher socioeconomic disadvantage were identified from areas with high populations of racial and ethnic minorities based on the CDC/Agency for Toxic Substances and Disease Registry Social Vulnerability Index [41]. After identifying the areas, Google searches were conducted to identify local barbershops and salons that were invited to participate in the study. Owners were contacted by phone to explain the study objectives and procedures, request permission for participation, and schedule an appointment to interview personnel. Barbers and stylists were eligible to participate if they were 18 years or older, had at least one year of work experience, and were willing and available for an interview. Purposive sampling of participants ensured the triangulation of perspectives and experiences by age, sex, venue, and county. The sample size for the interviews was determined based on data saturation [42], such that additional interviews would no longer yield new insights or themes relevant to the research questions [43]. A total of 16 participants were interviewed.

### 2.4. Research Team

The research team was predominantly African American (2 African American females, 2 African American males, 1 Asian American male, and 1 Caucasian female) to ensure cultural familiarity. Research assistants were trained in conducting in-depth interviews and maintaining ethical standards throughout the research process. Training sessions included instruction on qualitative research methods, interview techniques, ethical considerations, and data collection tools. Additionally, research assistants participated in mock interviews and received feedback to refine their skills before engaging with study participants. Research assistants were trained in transcription conventions specific to qualitative research to ensure data quality and accuracy.

### 2.5. Data Collection

Qualitative research assistants (PTM, SV, FS, and ND) visited each participating barbershop and salon and approached potential participants to introduce the study and assess their interest in participating in an in-depth interview. Informed consent was obtained from all participants. Face-to-face interviews were conducted in English in a private space at each participating establishment, allowing for the observation of context-specific characteristics that may inform intervention development and implementation. Two research assistants conducted each interview; one moderated and audiotaped the interview, asking questions and probing responses, while the other took field notes and observed for non-verbal cues. 

The sociodemographic data of all the participants were collected at the beginning of the interview. General introductory questions were asked to establish rapport and ensure the participants were comfortable before delving into more specific topics. The interview guide consisted of semi-structured questions related to community concerns, the impact of COVID-19, interest in health promotion and education, and prospective acceptability, including self-efficacy, perceived effectiveness, burden, and opportunity costs. The interview guide was developed and pilot-tested with a small sample of individuals in Richland County representing the target population to ensure the questions were clear, concise, and relevant to the study objectives. Feedback from pilot testing was used to refine and finalize the interview guide (see Supplementary Materials).

With participants’ consent, interviews were audio-recorded using a Sony ICD-PX370 mono digital voice recorder. Recordings were transcribed verbatim, then the transcripts were cross-checked against audio recordings by MK and PTM to identify and rectify any discrepancies and errors before being imported into NVivo 14 software (QSR International, Cambridge, MA, USA) for organization, coding, and the evaluation of themes [44]. Identifying information was removed from transcripts. Interview durations ranged from 12 to 47 min, with a mean of 32 min. Participants received a $50 gift card as compensation for time spent in the interview.

### 2.6. Analysis

A deductive coding approach was used to explore the data during coding by applying predefined codes derived from the TFA constructs [26]. This approach allowed for capturing themes and nuances in participant responses. The TFA comprises seven constructs (Table 1): affective attitude, burden, ethicality, intervention coherence, opportunity costs, perceived effectiveness, and self-efficacy [26]. The domain of intervention coherence was considered less applicable to the study objectives and was therefore not assessed. 

Thematic data analysis was guided by the Framework Method, which outlines seven steps to develop structured outputs that summarize data when multiple researchers are involved [45]. The steps are transcription, familiarization with the interview, coding, creating a working analytical framework, applying the framework, data charting, and interpretation [45]. Qualitative research assistants (SV, FS, ND) transcribed all audio recordings of the interviews verbatim (two research assistants per interview) into a Microsoft Word document. One of the authors, PTM reconciled each transcript by cross-checking with the original audio recordings and contextual field notes before rectifying the discrepancies or errors. Finalized deidentified transcripts were imported into NVivo software for coding.

PTM and MK then familiarized themselves with the data by reading through all the transcripts multiple times, taking notes, and highlighting key themes, concepts, and interesting quotes. PTM and MK independently conducted the initial coding of the 16 transcripts to identify key concepts and emerging themes in the data. The process was iterative. First, we identified the ideas and concepts and used them as codes. Then, we organized the codes into broader themes by identifying underlying patterns and constructs. The relationship between constructs was explored through discussion to create themes. Deductive codes were used to develop themes. Initial codes were organized into a working analytical framework that included broader categories of the codes derived from the TFA. The working analytical framework was systematically applied to the rest of the data, ensuring consistency in coding across all the transcripts. A matrix was created to organize coded data according to the TFA theme. PTM and MK analyzed the data within each theme to identify patterns and note any outliers or unexpected findings. Findings were summarized based on the TFA constructs. The Standards for Reporting Qualitative Research guidelines were used to report our findings [46].

### 2.7. Ethical Considerations

This study was deemed exempt from review by the University of South Carolina Institutional Review Board (Date: 7 July 2023, Number: Pro00129899). Permission to conduct qualitative interviews with personnel was sought from the barbershop and salon owners. All participants provided informed consent before the interviews.

## 3. Results

### 3.1. Participant Characteristics

Sixteen African Americans working full-time in barbershops or salons in Lexington, Orangeburg, Richland, and Sumter counties participated in the in-depth interviews (Table 2). Most participants were aged 20–39 years (*n* = 9), female (*n* = 9), and had completed some college or higher education (*n* = 11). Most professionals were seasoned, with nine of them reporting six or more years of experience in the profession (*n* = 9). Barbershops and salons were equally represented (*n* = 8 each).

### 3.2. Perceptions on the Acceptability of a COVID-19 Prevention Intervention

Participant reflections on the potential acceptability of delivering a COVID-19 prevention intervention were categorized based on the TFA constructs. Findings were described by the TFA construct and supported by illustrative verbatim quotes with minimal identifying characteristics (profession and age).

#### 3.2.1. Affective Attitude

Overall, participants expressed overwhelmingly positive affective attitudes towards delivering interventions to promote COVID-19 testing and vaccination within their professional settings. They highlighted their community influence and the trust placed in them by their clients and other community members. This trust extended beyond haircare to broader personal and community issues, positioning barbers and stylists as influential community figures capable of effectively promoting health interventions. The participants highlighted how the unique trust made them feel similar to healthcare professionals and counselors.

*“I think they (clients) are more so likely to listen, coming from somebody they follow and look up to.”*

(Barber, 34 years old)

*“I think we could play a big role in it and that people because they trust us with their hair and not just hair.”*

(Stylist, 32 years old)

*“We’re literally like doctors and psychologists because you take it, we’re literally the closest thing to the membrane. We’re right up on you.”*

(Barber, 50 years old)

*“A lot of my clients, they come to vent and be more like a counselor, listen to their problems, you know, try to encourage them to do the right thing, you know, that as well.”*

(Barber, 39 years old)

A few participants also acknowledged the persistent challenges COVID-19 posed to their communities. They expressed concern about the ongoing threat of COVID-19 in the community. Although several participants acknowledged the persistent challenge of COVID-19 in their community and expressed concern, several others also pointed to the decline in public concern for COVID-19. They noted that the frequency of discussion about the virus had decreased over time. Some participants also believed that COVID-19 had become a minor issue similar to the flu.

*“We talk about it, you know. COVID is still out there, and it’s still a big part of the salon and community settings.”*

(Stylist, 32 years old)

*“I can tell you right now the pandemic is scary, and when I say scary, because it’s not going away.”*

(Stylist, 66 years old)

*“Nobody has been talking about COVID lately.”*

(Barber, 34 years old)

*“When it was like out there like that when it was mandatory, we talked a lot about it, but now it’s like it’s died down. Nobody’s really concerned about it anymore.”*

(Stylist, 34 years old)

Other participants observed a difference in the uptake of COVID-19 vaccination by age. There was a general sentiment that younger people tended not to get vaccinated due to low perceived risk, while older people got vaccinated due to perceived high risk.

*“I feel like most of the older people are getting the vaccine more than the young people because they’re scared to get it (COVID-19) cause I’m honestly one of them (young people).”*

(Barber, 21 years old)

*“So, they (young people) are just naive, you know, thinking that they’re not gonna get it because they’re young.”*

(Barber, 39 years old)

#### 3.2.2. Self-Efficacy

Overall, participants’ confidence in their ability to deliver a COVID-19 prevention intervention varied widely, influenced by their confidence, knowledge, and skills to engage their clients effectively. Several participants felt confident and comfortable discussing COVID-19 with their clients and integrating these discussions into their routine conversations. Their knowledge and skills, dedication to the community, and shared experiences with loss and grief due to COVID-19 often reinforced this confidence.

*“I’m pretty good at, at setting something out and getting things done. So, if I was to be involved in something, I want to see it through, and I think my clients and staff would be willing to do it.”*

(Stylist, 32 years old)

*“I’m a seller… I can talk people into than anything”*

(Stylist, 45 years old)

*“Matter of fact, we talk about that a lot, you know, a lot of my clients have lost relatives as well as me, and I’ve lost a relative.”*

(Barber, 39 years old)

Conversely, some participants expressed reservations about their knowledge and ability to effectively convey information about COVID-19. These participants acknowledged gaps in their understanding of the virus and its prevention, which they felt could hinder their effectiveness. Despite these concerns, there was a willingness to learn and a recognition of the importance of accurate information dissemination.

*“I’m not real educated on COVID. I basically know what you know; most people know, you usually wear a mask and six feet at a distance. So, I would say probably a 5 out of 10.”*

(Barber, 33 years old)

*“I don’t even know if you can avoid getting it because it’s airborne, right?”*

(Barber, 34 years old)

#### 3.2.3. Perceived Effectiveness

Overall, participants strongly believed in the potential effectiveness of a COVID-19 prevention intervention. They highlighted several ways the intervention could positively impact their communities, emphasizing the dissemination of information, prevention of disease spread, and overall safety in the work environment and community.

Participants believed that discussing COVID-19 within their professional setting would have a ripple effect due to the high volume of clients they interact with daily, reaching a broader audience as clients share the information with others. This word-of-mouth approach was perceived as a powerful tool for raising awareness about COVID-19 and its long-term effects, encouraging regular testing and promoting vaccination.

*“The more we talk about it, you know, it goes further because you talk to one person, they talk to another person, you know.”*

(Barber, 28 years old)

*“We can spread the word pretty quickly because we got different people coming in.”*

(Barber, 49 years old)

Participants noted that educating clients about the ongoing risks of COVID-19 and the benefits of preventive measures could lead to broader community health improvements. They believed such interventions would reduce the spread of the disease and contribute to a safer community environment.

*“They could find out whether they have it or not, you know, and stop the spread of the disease.”*

(Barber, 39 years old)

*“I think it would be great just to push awareness, you know, and then to a lot of people are embarrassed so they may not want to go to the health department to go get a test because whatever you embarrassed for you, you know what I’m saying? But if you could come in and get a little kit going about your business, I think that more people would go ahead and get tested.”*

(Stylist, 46 years old)

#### 3.2.4. Ethicality

Overall, participants felt that their involvement in a COVID-19 prevention intervention was ethically appropriate and aligned with their sense of social responsibility. They recognized the critical need for accurate information about COVID-19 testing and vaccination in their communities and felt compelled to address this need.

*“A lot of them don’t have the information on the importance of COVID-19 testing and vaccination.”*

(Barber, 39 years old)

*“We do a lot of community service.”*

(Barber, 34 years old)

*“I think anything to educate the public in any form or fashion when it comes to COVID is a good thing.”*

(Stylist, 66 years old)

Nevertheless, vaccine hesitancy was substantial for the study participants and perceived as high among the clients. This skepticism was rooted in historical mistrust towards governmental and medical authorities, fueled by a broader skepticism of the government. Historical instances of unethical medical testing targeting minority populations were frequently cited as reasons for this mistrust. Participants shared a range of concerns about the rapid development of the vaccine, its safety, and effectiveness. In some instances, participants feared being used as “test dummies” for experimental treatments. Several participants noted that they had not been vaccinated because of mistrust in the government. Other reasons cited for non-vaccination among participants and in the community included side effects and concerns about the efficacy of the vaccine.

*“I just feel like they’re using us as test dummies, you know, taking the COVID shot. The masses don’t know what’s going on. I think they’re trying to decrease the population honestly.”*

(Barber, 49 years old)

*“People are hesitant to get a COVID shot here in the South because they’ve always tested on people, and a lot of people have died because they’ve tested. It’s not that we don’t trust medicine as we use medicine for everything, but I think that’s, it’s who wants to be somebody’s lab rat?”*

(Barber, 32 years old)

*“I know people who got it and a booster, they still caught it. I don’t know, maybe they got the wrong one.*

(Barber, 33 years old)

Some participants noted that some people were only vaccinated because of mandates at work or school. Inconsistent and confusing messages from health authorities have eroded confidence in the COVID-19 prevention interventions. Participants mentioned changing guidelines and conflicting information as factors that further eroded their trust. Some believed that the pandemic and vaccine were being used for population control or other nefarious purposes, reflecting a profound mistrust in public health messaging.

*“I know some people did it because they were forced to, you know, they didn’t want to lose their job or they didn’t, they wanted to go to school”*

(Salon, 46 years old)

*“The more that I watched the health department and Doctor Fauci every other day they were saying something different… So, I didn’t really trust what they were saying.”*

(Stylist, 46 years old)

#### 3.2.5. Burden

Most participants did not view delivering a COVID-19 prevention intervention as burdensome. They felt that the intervention could be seamlessly integrated into their existing client interactions without requiring additional effort or disrupting their routines. Participants noted that conversations about COVID-19 were already occurring due to the pandemic’s impact on their communities.

*“Matter of fact, we talk about that a lot, you know, a lot of my clients have lost relatives as well as me, I’ve lost a relative.”*

(Barber, 39 years old)

A few participants indicated concerns about the practical burdens of participating in additional training or delivering health interventions. Time constraints and the intensity of their work schedules were cited as potential barriers to engaging in in-depth conversations with clients during peak times. However, participants reported how the interventions fit into their normal work routine, as the clients could pick the self-test kits from a strategic location within the barbershop or salon. Also, health education would be disseminated during the haircutting or styling process.

*“I have to talk fast because when they come, they come, you know, it won’t be like how we are sitting down now talking then cut your hair when they come, they come.”*

(Barber, 34 years old)

*“Y’all are basically making it easy for us to go out and spread each other. I mean, we don’t have to go out. They come in here when they come in here, we can do work with them right now.”*

(Barber, 50 years old)

#### 3.2.6. Opportunity Costs

Many participants expressed willingness to participate; however, their ability to engage in training and intervention delivery was heavily influenced by the opportunity costs involved. Several participants highlighted that time constraints would significantly hinder their participation in additional training and intervention delivery, detracting from their primary business activities.

*“I can’t (participate in training). Not because I wouldn’t want to but because [mumbling] time of span.”*

(Stylist, 77 years old)

The concept of mutual benefit was emphasized, as participants needed to balance their professional responsibilities with their desire to contribute to community health. Financial incentives were viewed as a practical solution to offset the opportunity costs and support their engagement in the intervention.

*“It’s a scratch your back, scratch my back type of thing.”*

(Stylist, 59 years old)

*“I will be interested in the training depending on the length of time they will be able to take on the training. It will depend on incentives. Of course, getting paid for your time is always the best incentive.”*

(Barber, 32 years old)

## 4. Discussion

This study sought to assess barbers’ and stylists’ willingness to participate, attitudes, self-efficacy, perceived effectiveness, burden, and opportunity costs associated with interventions aimed at increasing COVID-19 testing and vaccination. The results indicated that delivering these interventions was generally acceptable. High acceptability was primarily due to the strong community influence and trust, the expected positive benefits of the intervention, and the minimal burden on participants. Despite a noted decline in public concern, the ongoing recognition of the public health threat of COVID-19 among participants and historical mistrust in the government, health authorities, and interventions in the United States supports the continued relevance of prevention efforts.

Participants highlighted their strong community influence and trust within the community, which could enhance the acceptability and impact of community-based health interventions. Studies have shown that trusted community figures can effectively disseminate health information and influence health behaviors due to their established relationships and credibility within the community [14,47]. Consistent with previous research, barbers and stylists viewed themselves as community role models, counselors, and problem-solvers, recognizing their potential to influence health behaviors beyond their traditional roles [27,48,49,50]. Leveraging this trust and influence can facilitate open and honest conversations about COVID-19, effectively disseminating accurate health information to address vaccine hesitancy, dispel misinformation, and promote COVID-19 prevention measures [16,51,52]. Previous findings show that interventions delivered in barbershops and salons can achieve high levels of engagement and acceptance, particularly among marginalized groups [16,17,18,19,20,21,22,23,24,25]. However, our study extends the literature by highlighting the potential challenges and opportunities in the context of a pandemic. 

Participants expressed strong beliefs in the potential effectiveness of the intervention, highlighting the perceived ability to reach a broad audience through word-of-mouth, prevention of disease spread, and overall safe environment. This aligns with the principles of social diffusion theory, which posits that behaviors and norms can be propagated through social networks [53]. The literature has shown the effectiveness of community-based information dissemination health programs delivered by barbers [54]. Similar interventions in other contexts have demonstrated that utilizing existing social networks can enhance the dissemination of health information and encourage preventive behaviors [54,55,56,57]. The potential for a ripple effect, where informed clients further spread the message, underscores the strategic value of partnering with barbers and stylists in public health efforts, especially in tightly knit African American communities. 

In terms of opportunity costs and burden, participants perceived delivering a COVID-19 prevention intervention as not overly burdensome, suggesting that such interventions can be integrated into existing practices with minimal disruption. Given that COVID-19 discussions are already occurring, the minimal perceived burden of integrating COVID-19 prevention discussions into routine client interactions about personal and health issues is a positive indicator of intervention feasibility. Previous studies have highlighted that interventions requiring minimal additional effort from implementers are more likely to be adopted and sustained [58,59,60]. 

While participants were willing to participate in a COVID-19 prevention intervention, reduced public concern, vaccine hesitancy, insufficient knowledge, and opportunity costs emerged as potential barriers. The observed decline in public concern about COVID-19 poses a challenge to the intervention’s acceptability. This trend has been documented in other studies, where the initial high levels of concern and uptake of preventive measures have declined over time [61,62]. Public health strategies must address this complacency by reinforcing the ongoing risks and the importance of preventive measures. This is particularly essential given the recent spike in COVID-19 infections in 36 states, including South Carolina [63].

Self-efficacy in intervention delivery varied based on perceived competence, confidence, and comfort. Some participants expressed a high level of comfort and confidence in their ability to engage in the intervention; others recognized that their current knowledge about COVID-19 was insufficient to address potential client questions and counteract false information. This COVID-19 knowledge gap was exacerbated by misinformation and confusing public health messaging and communication. Several studies have demonstrated that the social and public health impact of misinformation is more pronounced in African American communities, fueled by distrust in the government and health systems [64,65]. This distrust stems from past unethical medical practices and ongoing disparities in healthcare access and treatment [66]. Mistrust originating from historical deception and mistreatment by authorities and socioeconomic and health system disparities significantly pose attitudinal barriers to participation in health interventions. Strategies to reduce mistrust, starting with trusted members of the communities, can help increase participation among minority populations. Relatedly, vaccine hesitancy emerged as a significant barrier to vaccine uptake. Similar to the studies exploring contributors to COVID-19 vaccine hesitancy [67,68], participants expressed skepticism about the COVID-19 vaccine due to concerns about the side effects, safety, efficacy, and rapid development of the vaccines. This skepticism may make the acceptability of COVID-19 prevention interventions challenging, underscoring the need for culturally sensitive and trustworthy communication strategies to address vaccine hesitancy. 

Recent studies during the COVID-19 pandemic have highlighted the role of barbers and stylists in disseminating accurate information about COVID-19 testing and vaccination, including their potential to address vaccine hesitancy and misinformation, given their established rapport with clients [8,24]. Addressing vaccine hesitancy-related concerns will increase intervention acceptability and uptake and ensure the success of COVID-19 prevention interventions. Participants’ willingness to learn and improve their knowledge is promising and suggests that, with accurate information and adequate support, they can become effective health advocates. Comprehensive training will enhance the participants’ knowledge and confidence in discussing COVID-19, equipping them with communication skills and strategies for managing vaccine hesitancy and misinformation [69]. However, concerns about opportunity costs and time constraints to participate in training and intervention delivery highlight the need for practical solutions to support participation. Providing the appropriate incentives to acknowledge the time and effort of barbers and stylists and flexible training schedules could help mitigate the opportunity costs and encourage ongoing engagement in public health activities. Programs that use incentives have shown improved attendance and adoption of health behaviors [70,71]. 

The use of qualitative interviews allowed for a deep exploration of the perspectives, attitudes, and experiences of barbers and stylists regarding the COVID-19 intervention. The study included a diverse group of barbers and stylists from various backgrounds, enhancing the robustness of the findings and ensuring the representation of multiple viewpoints and experiences. Using the TFA provided a theoretical grounding to understand the multifaceted aspects of intervention acceptability, adding rigor and depth to the analysis. Guided by the findings of this study, we conducted a quantitative survey among barbers and hair salon owners, employees, and their clients, and the findings of the study will be published elsewhere. 

Despite these strengths, several limitations are worth noting. The qualitative nature of the study, while providing depth, may affect the generalizability of the findings to the broader population of barbers and stylists, especially those with different demographic or cultural characteristics. The experiences and perspectives of barbers and stylists can vary widely based on factors such as the size and location of their business, clientele, and personal attitudes toward COVID-19. This variability can make it challenging to draw broad conclusions. As with any qualitative study, there is a risk of interviewer and respondent biases. Participants may provide socially desirable responses, and interviewers may inadvertently influence responses through their questions or demeanor. Lastly, our study explored the prospective acceptability of a COVID-19 prevention intervention in barbershops and salons from the perspective of barbers and stylists; therefore, we cannot infer the acceptability or effectiveness of barbershop- and salon-delivered interventions from the perspective of clients. Additionally, as we considered prospective acceptability, the views of barbers and stylists may not reflect actual barriers and facilitators.

## 5. Conclusions

In conclusion, this study offers valuable insights into the delivery of COVID-19 interventions aimed at promoting testing and vaccination uptake from the perspective of barbers and stylists. The high acceptability of these interventions was primarily driven by community influence and trust, the anticipated positive benefits of the interventions, the minimal burden on participants, and the ongoing relevance of COVID-19 as a public health threat. Although leveraging the influence of trusted community figures shows substantial promise in driving meaningful behavior change and contributing to broader public health efforts, strategies to address vaccine hesitancy, declining public concern about COVID-19, and knowledge gaps remain crucial. Despite the low public concern about COVID-19, the recent substantial increase in COVID-19 cases in the summer of 2024 [63] demonstrates why this work is even more relevant now.

## Figures and Tables

**Table 1:** Operationalizing the Constructs of the Theoretical Framework of Acceptability.

TFA Construct	Original Definition [26]	Definition as Applied to This Study
Affective Attitude	How an individual feels about the intervention	How barbers and stylists feel about disbursing COVID-19 self-test kits and disseminating testing and vaccination information
Burden	The perceived amount of effort required to participate in the intervention	The effort required by barbers and stylists to integrate COVID-19 testing and vaccination information into their routine work
Opportunity Costs	The extent to which benefits, profits, or values must be given up engaging in the intervention	The time and financial costs barbers and stylists incur by participating in the COVID-19 intervention activities
Perceived Effectiveness	The extent to which the intervention is perceived as likely to achieve its purpose	Barbers’ and stylists’ beliefs about the potential effectiveness of the intervention in promoting COVID-19 testing and vaccination
Self-Efficacy	The participant’s confidence that they can perform the behavior(s) required to participate in the intervention	The confidence of barbers and stylists in their ability to effectively communicate and distribute COVID-19-related information and test kits

**Table 2:** Participant Sociodemographic Characteristics (N = 16).

Characteristics	*n*
Age group (years)	
20–39	9
40 or more	7
Sex	
Male	7
Female	9
Venue	
Barbershop	8
Salon	8
County	
Lexington	3
Orangeburg	2
Richland	7
Sumter	4
Highest level of education	
Did not graduate high school	1
High school diploma or GED	4
Some college or higher	11
Experience in profession (years)	
1–5	7
6–10	2
11 or more	7

## Data Availability

The original contributions presented in the study are included in the article, and further inquiries can be directed to the corresponding author.

## Supplementary Materials

The following supporting information can be downloaded at: https://www.mdpi.com/article/10.3390/vaccines12091011/s1, Table S1: Interview Guide.
