# Supplementary material for: Exploring the Views of Barbers and Stylists on the Acceptability of Delivering Community-Based Interventions to Promote COVID-19 Testing and Vaccination in South Carolina
Source: Vaccines (Basel). 2024 Sep 3;12(9):1011. doi: 10.3390/vaccines12091011 (PMC11436097; doi:10.3390/vaccines12091011)
Supplement: Supplementary file 1 [file vaccines-12-01011-s001.zip › vaccines-3117767-supplementary.pdf]

In-depth Interview Guide for Barbers and Stylists

**General Information about Participant, Business and Clients**
I want to start the interview by first talking about you, your business, and clients
Tell me about yourself.
Tell me a little about your clients.
Can you describe the kind of interactions you usually have with your clients?
**Interest in health promotion and education**
How important is health to you? What kinds of things do you do to stay healthy?
What do you think are the main health needs and concerns of your clients?
How do you feel about talking to your clients about health-related topics?
What role can barbers/stylists play in promoting health?
How likely would you be to support COVID-19 at home testing kit distribution? Health education on COVID-19 testing and vaccination?
What factors are considered when deciding what programs to implement in your business?
What information and evidence do you typically need when considering implementing a new program?
What do you rely on as sources of information for this evidence?
**Impact of COVID-19 on business, clients, and community?**
How has the COVID-19 pandemic impacted you personally?
How has the COVID-19 pandemic impacted your work?
How has the COVID-19 pandemic impacted your clients? And your community??
What would you say is the level of knowledge about COVID-19 in your community?
Barriers and Facilitators to COVID-19 Testing and Vaccination
Describe the general perceptions of COVID-19 vaccination and testing in your community?
If a client were to share their concerns about COVID-19 vaccination, how confident are you talking to them about the benefits of vaccination?
If you had to produce some ideas about what we could do to promote COVID-19 testing and vaccination in your barbershop/salon, what ideas do you have? There are no right or wrong answers, and we want you to think as creatively as you can to help us figure out what would work in barbershops and salons to get more community members to test and vaccinate against COVID-19 and improve the uptake of public health programs.
Project
Provide brief description of project
If you were to be involved in this project, how would you be able to assist barbershops and salons in your community to promote health, given your position and experience?
How interested do you think your clients would be in participating in the program?
Would you be available to participate in a one-day training to implement the program?
Who are the key individuals that would help the project to be successful?
What factors would help maintain a program like this? e.g. financial support, community partner, etc.
Who else should we speak with that may be knowledgeable about barbershops, salons and the community and willing to share their opinions with us?
Is the anything more you would want to tell me about what we have discussed today?
